# Early growth vigor monitoring and salt tolerance assessment of cotton based on SDI-YOLO11-seg

**DOI:** 10.3389/fpls.2026.1905593

**Published:** 2026-10-22

**Authors:** Deyi Lei, Zhibo Zhong, Ping Zhang, Jianling Guo, Liang Tang, Yu Yu, Xiuqing Fu

**Affiliations:** 1 College of Engineering, Nanjing Agricultural University, Nanjing, China; 2 Institute of Farmland Irrigation and Water Conservancy & Soil and Fertilizer, Xinjiang Academy of Agricultural Reclamation Sciences, Shihezi, China; 3 Management Committee of Alar National Agricultural Science and Technology Park, Xinjiang Production and Construction Corps, Alar, China; 4 Key Laboratory for Crop System Analysis and Decision Making, National Engineering and Technology Center for Information Agriculture, Engineering Research Center of Smart Agriculture, Ministry of Education, Ministry of Agriculture, Nanjing Agricultural University, Nanjing, China; 5 Northwest Inland Region Key Laboratory of Cotton Biology and Genetic Breeding, Cotton Institute, Xinjiang Academy of Agricultural and Reclamation Science, Shihezi, China

**Keywords:** experimental study, full-time series vigor monitoring system, instance segmentation, salt tolerance assessment, Xinjiang cotton

## Abstract

By precisely quantifying the growth changes of seed radicles and cotyledons over continuous time periods, the impact of saline stress environments on early crop growth can be assessed. However, traditional manual measurements struggle to accurately quantify these indicators continuously. Therefore, we developed a Full-Time Series Crop Growth Vigor Monitoring System. This system captured images of the hydroponic germination process and the soil-based seedling growth process of Xinjiang cotton seeds. We constructed a labeled dataset for identifying the germination process and calculating cotyledon growth. Using the SDI-YOLO11-seg model, we achieved the extraction and quantification of germination rate, emergence rate, radicle length, and cotyledon area over continuous time, validating the feasibility of a rapid, batch-assessment method for early cotton seed growth vigor based on the full-time series monitoring system and deep learning. Building on this, we conducted experiments involving four Xinjiang cotton varieties under hydroponic (germination stage) and soil-based (seedling stage) conditions with six different concentrations of NaCl solution stress. Germination rate, emergence rate, radicle length, and cotyledon area for each variety were measured continuously over time. The results show that the proposed SDI-YOLO11-seg model achieved a mean average precision for bounding boxes at IoU 0.5 (mAPbox50) of 0.961 and for masks (mAPmask50) of 0.937, representing improvements of 0.9% and 6.0% over the baseline YOLO11-seg, respectively. Validation against manual ImageJ measurements yielded coefficients of determination (R²) of 0.970 for radicle length and 0.993 for cotyledon area, confirming high measurement accuracy. Under salt stress concentrations ranging from 0 to 250 mmol/L, Jinken 1565 exhibited the fastest germination rate, the highest final germination rate, and the longest average radicle length, indicating strong salt tolerance during the bud stage. In contrast, Jinken 1775 demonstrated a higher seedling emergence rate, the fastest cotyledon growth rate, and the largest cotyledon area, suggesting excellent salt tolerance during the seedling stage. The Tahe 2 variety exhibited weaker salt tolerance during both the bud and seedling stages compared to the other three varieties. Coefficient of variation analysis further revealed that Jinken 1565 exhibited the most uniform radicle growth under high salt stress during the germination stage, while Jinken 1775 and Xinluzao 64 demonstrated superior uniformity in seedling growth, complementing the mean-based vigor assessment.

## 1 Introduction

China is one of the world’s largest producers and consumers of cotton, with Xinjiang serving as its primary cotton-producing region. Xinjiang’s cotton output accounts for 8.0% of global production (Feng et al., 2024). In 2024, the cotton sowing area in Xinjiang reached 2.4479 million hectares, with a record-high yield of 5.686 million tons, constituting 92% of China’s total cotton production. However, as Xinjiang is located deep inland, far from the ocean, it is characterized by a typical temperate continental arid climate featuring scarce precipitation and intense evaporation, leading to widespread and severe soil salinization (Xiao et al., 2023). The Third National Land Resource Survey of Xinjiang revealed that 9.27% of the region’s existing cropland, covering 274.7 thousand hectares, is severely salinized. Soil salinization is likely to be further exacerbated by global climate change and alterations in land use patterns (Bi et al., 2024). Therefore, screening and promoting high-quality salt-tolerant cotton varieties can leverage these underutilized land resources, expand the cotton cultivation area, and unlock the land’s productive potential. This approach will better equip Xinjiang’s cotton industry to tackle the challenge of soil salinization and ensure the long-term stable development of the cotton sector.

Salt stress is recognized as one of the major threats to cotton production, making the breeding of cotton varieties that can adapt to saline-alkali environments a key research focus for agronomists. Salt tolerance is typically assessed during the seed germination and seedling growth stages, as these are the periods when cotton is most sensitive to salinity (Ibrahim, 2016; Li et al., 2023). Traditional screening methods for salt-tolerant cotton varieties include field screening in natural saline-alkali soils, screening in artificial salt beds, seed germination tests, seedling hydroponic experiments, and measurement of physiological and biochemical indicators (Xu et al., 2021; Li et al., 2020; Hussain et al., 2021; De Costa et al., 2012; Xie et al., 2024). The first two methods are categorized as field screening. While they offer high environmental authenticity and allow for comprehensive trait evaluation, they involve long experimental cycles and are susceptible to weather variability. The latter three methods are indoor screening approaches. They provide the advantages of controllable environmental conditions and facilitate detailed studies. However, indoor methods typically involve concentrated data analysis at specific stages of seed germination or seedling growth, and they require significant manual labor for tasks such as seed cultivation management and data collection.

Conventional indoor methods for assessing seed germination and growth vigor typically include observational counting, chemical assays, and environmental control techniques. These approaches rely on manual observation, reagent reactions, or instrumental assistance to determine seed vigor, and are associated with numerous drawbacks such as low efficiency, significant subjective error, lack of standardization, destructiveness, and susceptibility to environmental interference. To address these limitations, many researchers have begun applying machine vision and imaging technologies to the fields of crop germplasm screening and vigor detection. For instance, building upon YOLOv8, Tian developed the FSG-YOLOv8s object detection model specifically for monitoring tomato seed germination vigor, measuring the germination rate, germination potential, and germination index of tomato seeds under different cultivation conditions (Tian et al., 2025). Ouyang proposed a non-destructive method for the full-process germination monitoring of radish seeds based on the lightweight YOLOv8-R model, finding that low-concentration ZnO-NPs priming treatment could enhance the germination rate and germination potential of radish seeds under salt stress (Ouyang et al., 2024). Sha collected full-time series images of the licorice seed germination process and utilized the YOLOv8 detection model to assess the germination status under different salt stress conditions, enabling rapid identification of licorice seed salt tolerance (Sha et al., 2024). By integrating soybean germination process images with the YOLOv8-seg model, Wu calculated radicle contour features and length, elucidating the morphological evolution of radicle contours over the germination period (Wu et al., 2024). Wang utilized his independently developed SeedRanger semi-automatic high-throughput RGB imaging system to acquire images every 30 minutes under dark cultivation conditions at 28 °C, conducting continuous monitoring over 120 hours. Using computer vision algorithms, he extracted 17 dynamic image-based traits (i-Traits) and performed a multi-point time-course GWAS analysis incorporating whole-genome resequencing data from 356 materials. This approach identified 541 non-redundant genetic loci associated with seed viability, revealing the spatiotemporal genetic architecture underlying cotton seed viability (Wang et al., 2026). Li employed an improved RT-DETR model to detect cucumber radicles in a soil environment, extracting germination rates under different salt stress conditions (Li et al., 2024). Ng introduced a multi-view oil palm seed quality classification model based on deep learning image matching technology, utilizing multi-view images to simultaneously process large batches of germinating oil palm seeds to predict the quality of each seed (Ng et al., 2024). Duc presented a method for predicting the hundred-seed weight of soybeans using seed structural traits and machine learning models, extracting eight structural traits from soybean seeds by combining machine learning models with RGB images (Duc et al., 2023). Colmer developed a phenotyping platform named SeedGerm, which achieved high-throughput, high-precision seed germination phenotyping analysis through automated imaging and machine learning (Colmer et al., 2020). Evidently, compared to traditional data acquisition methods, machine vision and imaging technologies in seed testing and agricultural data collection offer advantages such as high-throughput acquisition, batch processing, non-contact analysis, and data consistency. Furthermore, they enable high-efficiency and high-precision data analysis, significantly reducing labor costs (Kamilaris and Prenafeta-Boldú, 2018; Singh et al., 2021; Murphy et al., 2024).

Therefore, this study integrates machine vision and image analysis technology to collect full-time series growth images of cotton during the germination and seedling stages under saline stress conditions. It utilizes deep learning algorithm models to extract phenotypic features from the images, analyzing vigor indicators such as radicle length after seed germination and cotyledon area of seedlings. This approach enables the rapid detection of seed germination and growth vigor under different salt stress levels. The research aims to identify salt tolerance during the early growth stages of different cotton varieties, thereby achieving the goal of screening salt-tolerant cultivars. In addition to evaluating the mean growth vigor indicators, this study also assesses the uniformity of varietal growth under salt stress using the coefficient of variation, thereby providing a more comprehensive evaluation of salt tolerance from both central tendency and dispersion perspectives.

## 2 Materials and methods

### 2.1 Development of the full-time series crop growth vigor monitoring system

To enable our deep learning model to possess strong generalization capability, we require a sufficient number of cotton early-growth images for model training. For this purpose, we developed a Full-Time Series Crop Growth Vigor Monitoring System. This system integrates multiple functions, including environmental control, seed germination cultivation, rail-based high-throughput image acquisition, and automated irrigation. It is designed to create a suitable indoor environment for cotton seed germination and growth while eliminating human interference during the image acquisition process. The entire system consists of six heating incubators, four cooling incubators, and one central control console. The heating incubators can regulate the internal temperature from room temperature up to 50 °C, while the cooling incubators, utilizing compressors, can control the internal temperature from 5 °C to room temperature. The central control console manages the on/off status and imaging parameters of each incubator. The specific system composition and functional principles are illustrated in **Figure 1**.

**Figure 1:**
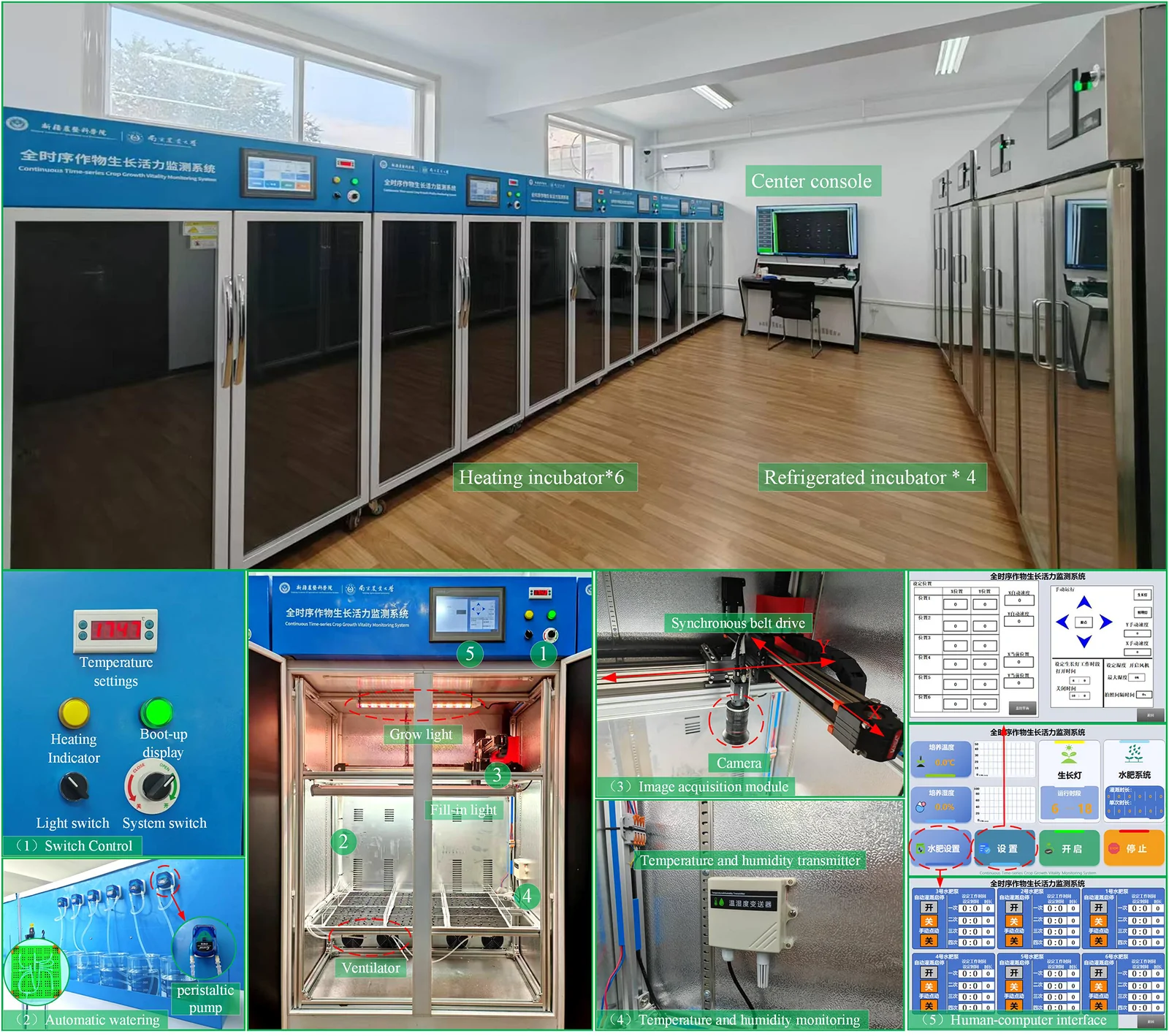
Full-time series crop growth vigor monitoring system.

Developed according to the requirements of standard germination experiments, the Full-Time Series Crop Growth Vigor Monitoring System can create the necessary environmental conditions—including moisture, temperature, air humidity, and light—for seed germination and subsequent seedling growth indoors. Simultaneously, it can automatically and continuously capture growth images of seeds from the germination stage to the seedling stage over successive time periods. The overall architecture of the system is illustrated in **Figure 2**. The environmental control module of this system utilizes temperature and humidity transmitters installed inside the chamber monitor real-time data at their respective locations, including temperature, humidity, and CO_2_ concentration. However, systematic spatial uniformity tests across the chamber volume have not yet been conducted. The current system relies on the forced-air circulation design (via PLC-controlled fans) to minimize spatial gradients, as described in Section 2.1. This allows for real-time monitoring and adjustment of the internal conditions. If the temperature falls below the set value, PTC heating panels are activated for heating; if the temperature is too high, the refrigeration compressor is activated for cooling. Concurrently, if air humidity or CO_2_ concentration exceeds the set thresholds, fans can be operated via PLC control to maintain suitable humidity for the seeds and prevent CO_2_ accumulation inside the chamber. The main body of the rail-based image acquisition module employs a synchronous belt transmission mechanism. A PLC program (FX3U-32MT/ES-A, Mitsubishi Electric, Dalian, China) controls a stepper motor (LC57HS64, Lichuan Electric, Lianyungang, China) to drive precise movement of the guide rail along the X and Y axes. This enables the mounted 20-megapixel RGB industrial camera (MV-CS200-10GC, Hikvision, Hangzhou, China) to automatically capture growth images of cotton seeds in six designated areas within the chamber. The captured images are then transmitted via a GigE Gigabit network high-speed interface to a PC for storage. The automatic irrigation module delivers solution from beakers to the bottom of the culture dishes via six peristaltic pumps located at the rear of the chamber, achieving timed and quantitative automated watering. The chamber was equipped with two types of lighting fixtures: (1) full-spectrum LED growth lights (model: YFS-5050-60, Shenzhen Yingfei Technology Co., Ltd., China; power: 18 W per strip; color temperature: 4000 K; photosynthetic photon flux density, PPFD: 150 ± 10 µmol·m^-2^·s^-1^ at 15 cm distance; spectral range: 380–780 nm); and (2) white LED fill lights (model: LT-3528-120, Shenzhen Leitu Optoelectronics Co., Ltd., China; power: 12 W per strip; color temperature: 6500 K; illuminance: 5000 ± 200 lux at 15 cm distance), simulating diurnal cycles and providing ample illumination for image capture by the camera. The human-machine interface (HMI) allows for the manual configuration of environmental parameters such as temperature, humidity, and light, as well as setting the time interval and area range for image acquisition. It should be noted that this system has not yet undergone a systematic spatial uniformity test across the entire incubator volume. However, to ensure the reliability and reproducibility of the experimental results, this study has implemented the following measures: (1) Three replicate petri dishes (for hydroponic experiments) and three replicate seedling trays (for soil-based experiments) were set up for each cultivar as biological replicates; (2) All experiments for each cultivar were conducted simultaneously under uniform environmental conditions to minimize batch-to-batch variability; (3) Image acquisition employed an automated procedure with a fixed trajectory path and preset time intervals to ensure consistent data acquisition conditions across all replicate areas. The precise spatial uniformity testing of the system will serve as an important component of subsequent research efforts.

**Figure 2:**
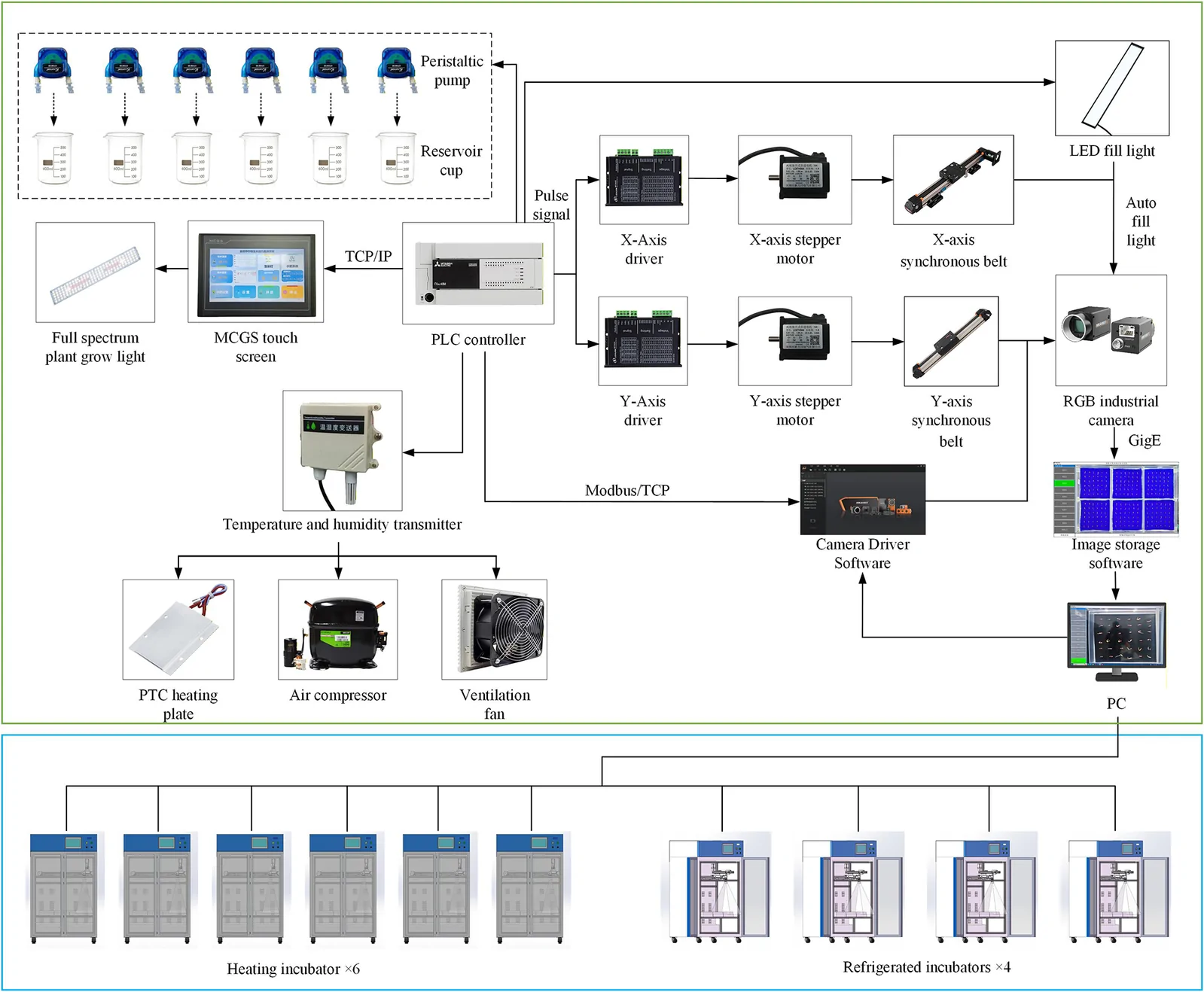
Overall architecture of the full-time series crop growth vigor monitoring system.

### 2.2 Image acquisition and dataset construction

Firstly, a preliminary experiment for the hydroponic germination stage and soil-based seedling stage of cotton was conducted at the Institute of Mechanical Equipment, Xinjiang Academy of Agricultural and Reclamation Sciences. Four cotton varieties—Jinken 1565, Jinken 1775, Tahe 2, and Xinluzao 64—provided by the Cotton Research Institute of Xinjiang Academy of Agricultural and Reclamation Sciences, were used. A 16-hour priming treatment was applied to the cotton seeds. For each variety, approximately 200 plump, undamaged, and delinted cotton seeds were selected. The seeds were surface-sterilized and their seed coats softened by soaking in a 5% sodium hypochlorite solution for 15 minutes. After rinsing three times with sterile water, a piece of A4-sized filter paper was moistened and laid flat horizontally on the lab bench. Thirty-six seeds were selected and arranged in two rows, starting 2 cm from the top edge of the filter paper, with 18 seeds per row and a 2 cm gap between the rows. All seeds were oriented with the micropyle facing downward to facilitate water absorption and radicle emergence. Subsequently, another moistened piece of filter paper was aligned and placed over the seeds, and the upper portion (about 1.5 cm from the top) was folded over to prevent water evaporation. The filter paper assembly was then folded and rolled horizontally into a cylindrical shape, with the end secured tightly using a rubber band. Finally, the bottom end of the filter paper roll was inserted into a beaker containing sterile water, and the setup was placed in an incubator for dark priming treatment, as shown in **Figure 3A**. After the 16-hour priming period, the filter paper roll was opened. Plump seeds were selected and arranged in a 6×6 grid in a culture dish, each Petri dish contains 36 seeds; groups of three Petri dishes represent the same cultivar; six Petri dishes are placed in each incubator; all cultivar trials are conducted under uniformly controlled environmental conditions to ensure experimental consistency. The culture dish was then placed into one of two heating incubators for a 48-hour germination test at a set temperature of 28 °C. During this period, a camera sequentially captured images at six designated positions, with a capture interval of 20 minutes per cycle. A total of 3, 456 germination images for the four Xinjiang cotton varieties were collected, as shown in **Figure 3B**. Following the completion of the hydroponic germination stage preliminary experiment, the seed priming steps were repeated. A nutrient substrate (containing vermiculite, coconut coir, and perlite) was thoroughly mixed with river sand (1–3 mm) at a 4:1 volume ratio to simulate the soil conditions for cotton seedling cultivation in Xinjiang. Plump seeds from the priming treatment were selected and placed in seedling trays in a 3×4 arrangement for seedling cultivation, each seedling tray contains 12 seeds; trays numbered 3–6 represent the same cultivar. Six seedling trays are placed in each incubator. All cultivar trials are conducted under uniformly controlled environmental conditions to ensure experimental consistency. The soil-based preliminary experiment lasted for 12 days, with an image capture interval of 30 minutes, resulting in the collection of 9, 216 cotyledon growth images, as shown in **Figure 3C**.

**Figure 3:**
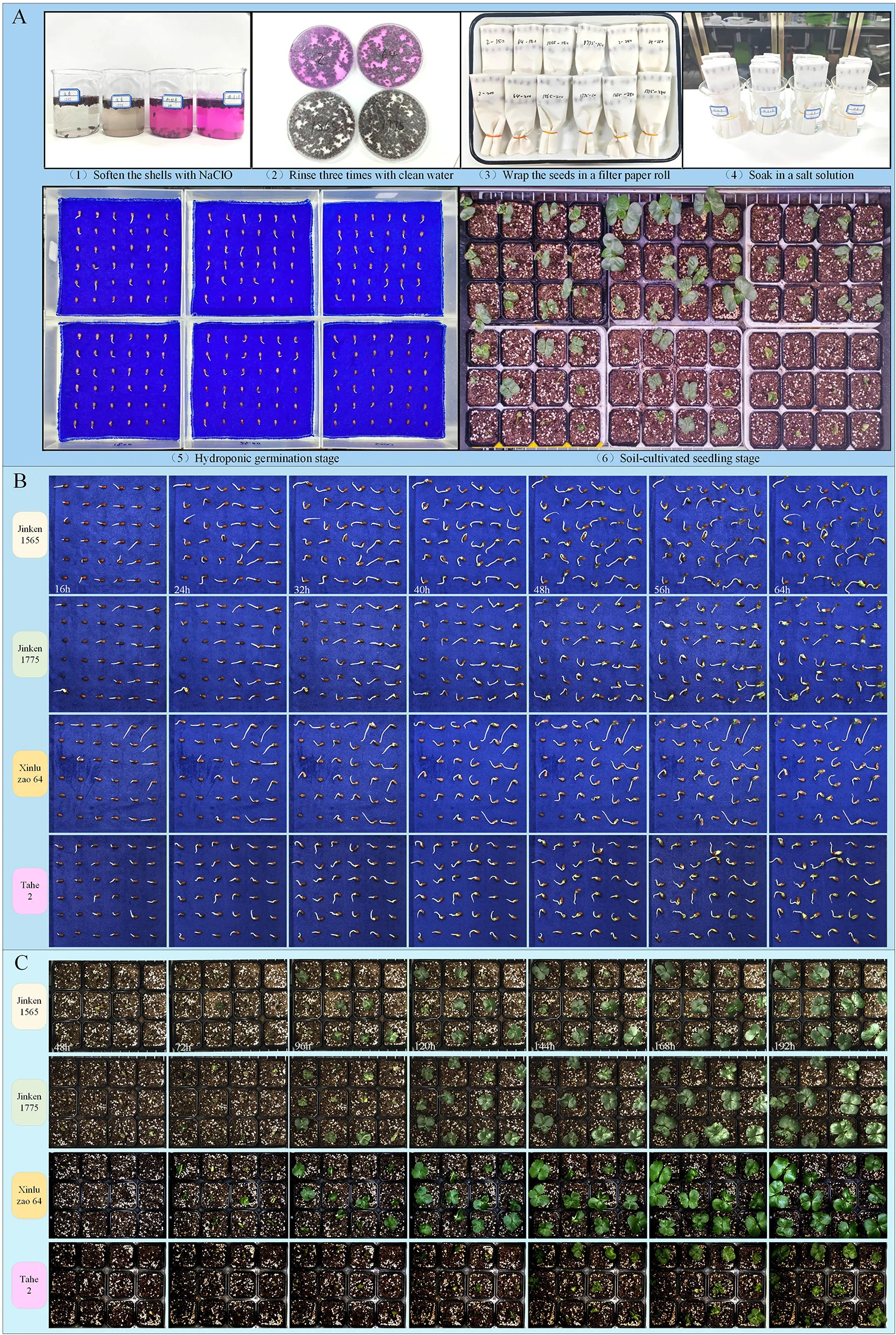
Image acquisition process: **(A)** pre-experiment procedure; **(B)** germination stage image acquisition; **(C)** seedling stage image acquisition.

From the total of 12, 672 raw images acquired during both the germination (3, 456 images) and seedling (9, 216 images) stages, we filtered out low-quality images caused by network fluctuations, camera shake, ambient light interference, and exposure failures. A total of 1, 200 high-quality images were retained, representing approximately 9.47% of the original dataset. These retained images comprehensively covered all growth postures of cotton seeds from the radicle tip emergence stage to the cotyledon expansion stage, ensuring sufficient diversity for model training despite the high rejection rate. Instance segmentation aims to achieve precise delineation of different object boundaries in an image through pixel-level classification (Rawat et al., 2022). To accomplish high-precision segmentation of multiple radicles within a single image, this study utilized the “Polygons” tool in the labelme software to perform multi-point closed annotations for the contour of each radicle. This ensures the model can capture the edge details of the radicle images. The annotated radicle and cotyledon regions were respectively labeled as “root” and “cotyledon”. Since the label files saved by the software are in JSON format, a Python script was employed to convert all label files into the TXT text format to meet the data specifications of the YOLO framework. Subsequently, multi-dimensional data perturbation strategies were employed to enhance model robustness. These included: non-uniform scaling to simulate multi-viewpoint scenarios, random rotation to simulate multi-angle scenarios, random brightness adjustment to simulate lighting variations, Gaussian blurring to replicate defocus phenomena, addition of Gaussian noise to simulate sensor noise, and contrast enhancement to optimize shadow details. Building upon the 1, 200 original images, the dataset was expanded to 2, 400 images using the methods described above. The samples were then partitioned in an 8:1:1 ratio into a training set (1, 920 images), a validation set (240 images), and a test set (240 images). The final composition of the dataset is presented in **Table 1**.

**Table 1:** Key parameters for preliminary soil-based cotton cultivation trials.

Enhancement	Root example	Cotyledon example	Number (sheets)	Format parameter	Proportion
Original image	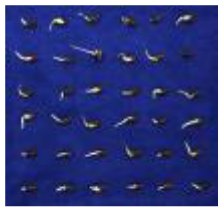	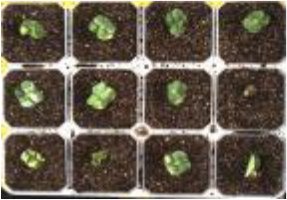	1200	JPG	50.00%
Random deflation	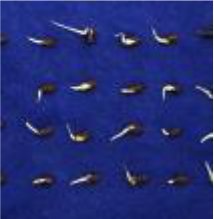	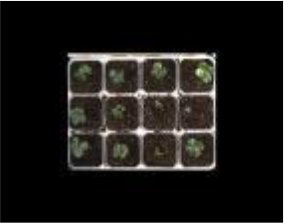	200	JPG Scale = (0.5, 1.5)	8.33%
Random rotation	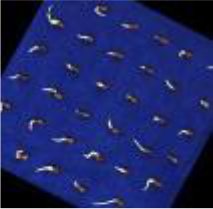	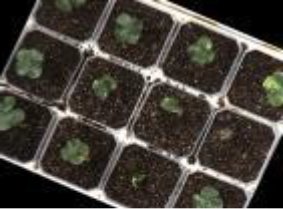	200	JPG Rotate = (-45°, 45°)	8.33%
Random brightness	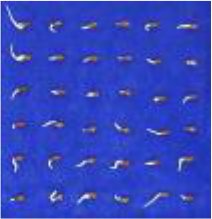	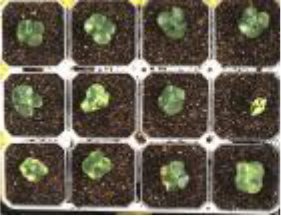	200	JPG Brightness = (-30, 30)	8.33%
Gaussian blur	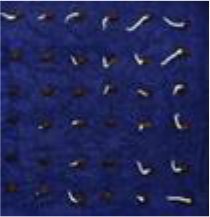	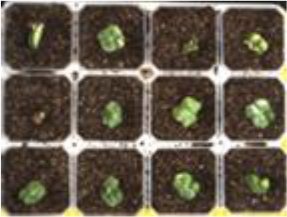	200	JPG Sigma = (0.0, 8.0)	8.33%
Gaussian noise	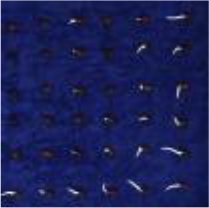	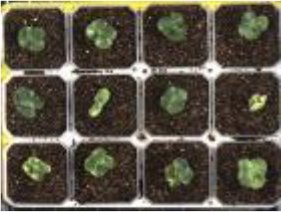	200	JPG α = (0, 0.5)	8.33%
Contrast ratio	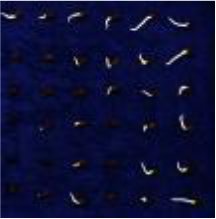	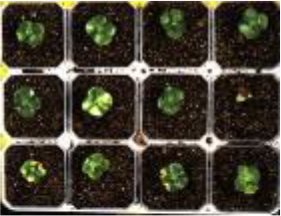	200	JPG Gamma = (0.5, 2.0)	8.33%

### 2.3 Selection of image segmentation model

Compared to traditional segmentation models, YOLO adopts a single-stage design that integrates object detection and instance segmentation into a unified workflow. This approach avoids the repeated feature extraction inherent in traditional two-stage models (e.g., Mask R-CNN). Through its anchor-free mechanism and dynamic optimization strategies, YOLO establishes significant advantages in speed-accuracy balance, multi-task compatibility, and generalization in open scenarios. Its lightweight nature makes it particularly suitable for high-precision requirements in resource-constrained environments (Liang et al., 2025; Koirala et al., 2019; Lawal, 2024). Numerous studies have applied YOLO-based instance segmentation models to various aspects of agriculture. For example, they have been used to estimate crop leaf area index (LAI), fruit maturity, and disease lesion area on leaves; to segment the peduncle-fruit connection region in real-time for millimeter-level positioning accuracy in automated harvesting systems; and for non-destructive, *in-situ* root image segmentation for root architecture screening (Sapkota et al., 2024; Wang et al., 2024; Yu et al., 2019; Yue et al., 2023). This paper will conduct a comparative analysis of five existing YOLO-series instance segmentation models. Their evaluation metrics under identical training configurations and datasets will be compared, and the model most suitable for cotton radicle and cotyledon detection and segmentation will be selected based on comprehensive performance.

Based on the Windows 11 operating system, a virtual environment for model training was established using the high-performance computing cloud service platform OpenBayes. The GPU used was an NVIDIA GeForce RTX 4090 (24 GB VRAM). The deep learning framework was constructed with Ultralytics 8.3.89, PyTorch 2.0.1, and Python 3.8.18. The hyperparameters for the model training in this study are detailed in **Table 2**.

**Table 2:** Main hyperparameters for training the object detection model.

Training configurations	Parameters
imgsz	640×640
epoch	100
batch	16
workers	8
momentum	0.937
close_mosaic	10
optimizer	SGD

The performance metrics of the five models under identical training conditions are recorded in **Table 3**. Among them, mAPbox50 and mAPmask50 are the core indicators for evaluating model performance, representing the precise localization capability for the detection task and the contour delineation ability for the segmentation task, respectively. Params and FLOPs denote the number of learnable parameters and the floating-point operations in the model, respectively, which are used to measure the model size and computational complexity. Higher values indicate a stronger ability to process complex data features, but correspondingly demand higher hardware requirements. FPS (frames per second) reflects the model’s processing efficiency per unit time, serving as a metric for real-time detection capability. The formulas for calculating mAPbox50 and mAPmask50 are shown below (Equation 1):

**Table 3:** Performance metrics comparison of different models.

Model	mAPbox 50	mAPmask 50	Params (M)	FLOPs (G)	FPS (f/s)
YOLOv5n-seg	0.951	0.865	**1.88**	6.70	128.16
YOLOv8n-seg	0.947	0.875	3.26	6.05	**157.43**
YOLOv9c-seg	**0.961**	**0.887**	27.62	79.54	99.97
YOLO11-seg	0.952	0.877	2.83	**5.18**	151.79
YOLO12-seg	0.949	0.871	2.81	5.20	121.61

Bold values indicate the best performance for each metric.

(1) $$\begin{array}{c} mAP\text{box}50=\frac{1}{N_{\text{cls}}}\sum_{c=1}^{N_{\text{cls}}}APc^{IoU=0.5}\\ mAP\text{mask}50=\frac{1}{N_{\text{cls}}}\sum_{c=1}^{N_{\text{cls}}}APc^{\text{mask}-IoU=0.5}\\ Precision=\frac{TP_{0.5}}{TP_{0.5}+FP_{0.5}}\\ Recall=\frac{TP_{0.5}}{TP_{0.5}+FN_{0.5}}\\ {AP}_{\text{c}}=\int_{0}^{1}P_{c}(R_{c})\text{d}R_{c}\\ mAP=\frac{1}{C}\sum_{c=1}^{C}AP_{c} \end{array}$$

To select the optimal baseline model for cotton radicle and cotyledon segmentation, we compared five state-of-the-art YOLO-series instance segmentation models (YOLOv5n-seg, YOLOv8n-seg, YOLOv9c-seg, YOLO11-seg, and YOLO12-seg) under identical training configurations and datasets (**Table 3**). As shown in **Table 3**, YOLOv9c-seg achieved the highest mAPbox50 (0.961) and mAPmask50 (0.887), but its parameters (27.62 M) and FLOPs (79.54 G) were approximately 10 times those of the other models, posing high computational demands for practical deployment. In contrast, YOLO11-seg achieved competitive mAPbox50 (0.952) and mAPmask50 (0.877) while maintaining low parameters (2.83 M), low FLOPs (5.18 G), and the highest FPS (151.79 f/s), demonstrating the best overall trade-off among accuracy, efficiency, and deployability. Therefore, we selected YOLO11-seg as the foundational model for this study.

It should be noted that the model comparison in this study primarily focuses on the single-stage instance segmentation models within the YOLO series. This choice is based on the following considerations: (1) The YOLO series has demonstrated an excellent accuracy–speed trade-off in real-time agricultural detection and segmentation tasks; its lightweight design is particularly well-suited to the deployment requirements of this system in resource-constrained environments; (2) Although the DETR series has achieved advanced accuracy levels in certain detection tasks, its Transformer-based architecture typically entails higher computational complexity and inference latency. While lightweight DETR variants such as RT-DETR have emerged in recent years, given the system’s real-time monitoring requirements and the feasibility of edge deployment, the YOLO series remains the more appropriate foundational choice at present.

### 2.4 Evaluation of the YOLO11-seg initial model

YOLO11-seg, as the latest instance segmentation model in the YOLO series officially released by Ultralytics, incorporates several groundbreaking improvements in algorithmic architecture, task adaptability, and performance optimization. Compared to previous versions, it achieves a synergistic breakthrough in accuracy and efficiency through innovations such as novel attention mechanisms (DSAM, SimAM Slice), structural lightweighting (C3k2, VanillaNet), and multi-scale dynamic optimization (SPPF_attention, dynamic receptive field) (Jin et al., 2025). Although the comprehensive performance of the YOLO11-seg model in cotton radicle detection and segmentation surpasses that of the other three models, its actual performance has not yet met the requirements for precise measurement of radicle length and cotyledon area, indicating significant room for improvement. **Figure 3** illustrates the workflow for extracting radicle length and cotyledon area at different growth stages from the captured images. First, the model is trained and the optimal weights are selected based on the self-built cotton germination and seedling growth dataset. Then, target images are chosen for inference to obtain instance segmentation results. Based on these segmentation results, masks for the radicle and cotyledon regions are extracted. Subsequently, the number of pixels within the mask contours and interior areas is measured. Finally, the actual length of each radicle and the actual area of the cotyledons are calculated separately using a scale factor. Therefore, the detection and segmentation accuracy of the model directly determines the precision and reliability of the root length and cotyledon area measurements. However, as shown by the instance segmentation results in **Figures 4**, **5**, the initial YOLO11-seg model exhibits some shortcomings in detecting and segmenting cotton radicles and cotyledons. In the early stages, a small number of missed detections occur (indicated by yellow circles). In the later germination stages, more severe instances of duplicate detection are present (indicated by pink circles). During the initial soil emergence of cotyledons, a few false detections are observed (indicated by red circles). In the segmentation stage, issues such as discontinuous segmentation regions and unsmooth segmentation edges arise due to misdetections (indicated by white circles). These phenomena can lead to a single radicle or cotyledon being misidentified as multiple objects, thereby affecting the measurement of vigor indicators during the germination and seedling stages. Consequently, to accurately measure the salt tolerance of the four cotton varieties during the germination and cotyledon stages using the model, corresponding improvements to the initial YOLO11-seg model are necessary.

**Figure 4:**
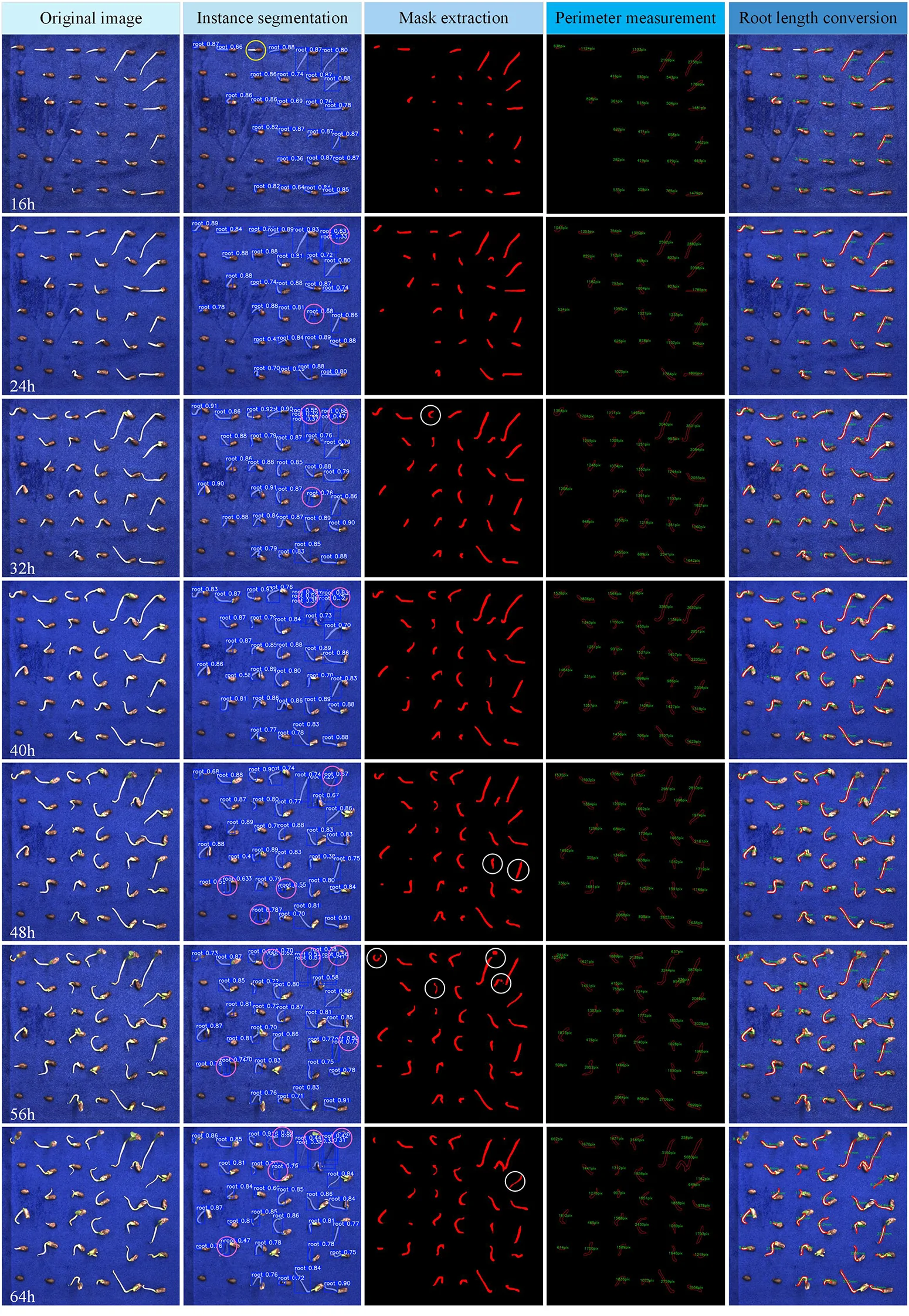
Radicle length measurement based on YOLO11-seg.

**Figure 5:**
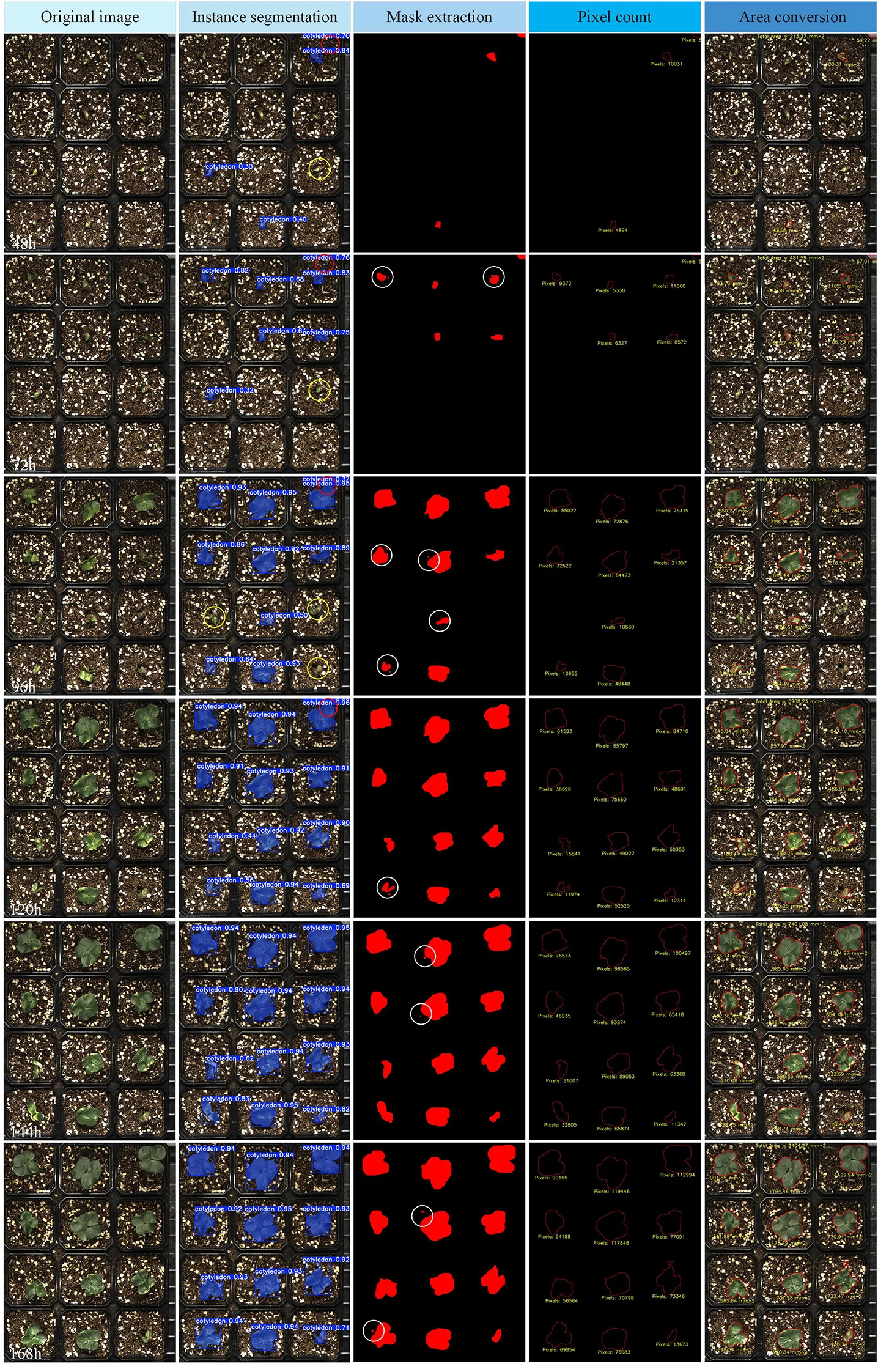
Cotyledon area measurement based on YOLO11-seg.

### 2.5 Improvement scheme for the YOLO11-seg model

To further enhance the model’s detection and segmentation accuracy, and to mitigate issues such as missed detections, duplicate detections, and discontinuous masks, the following improvements were made based on YOLO11-seg:

1. Introduction of a Tiny Object Detection Head: Due to the model’s high rate of missed detections for small radicles in the early germination stage of cotton seeds, a tiny object detection head was added to boost the model’s capability for detecting small targets. This equips the model with four detection heads (P2, P3, P4, P5), enabling it to detect targets as small as 4×4 pixels and strengthening its ability to detect cotton radicles at multiple scales (Bochkovskiy et al., 2020; Wu et al., 2023), as shown in **Figures 6A, B**.
2. Replacement with Dynamic Upsampler DySample: By substituting the original nn.Upsample in the Neck layer with DySample, the model’s multi-scale feature fusion capability is optimized, which is particularly beneficial for small object detection. Furthermore, DySample has been shown to enhance instance segmentation performance in multiple dense prediction tasks, improving edge smoothness and preserving edge details in segmentation tasks (Liu et al., 2023), as shown in **Figure 6C**.
3. IoU Optimization: IoU optimization was performed by introducing Inner-IoU, primarily addressing the limitations of traditional IoU in bounding box regression and enhancing model performance across multiple dimensions, thereby improving the model’s generalization capability (Zhang et al., 2023). Inner-IoU significantly improves small object detection through a multi-scale auxiliary bounding box generation mechanism. By flexibly adjusting the scaling factor ratio, the model can adapt to both dense radicle and sparse cotyledon scenarios, reducing the rate of missed detections, as shown in **Figure 6D**.

**Figure 6:**
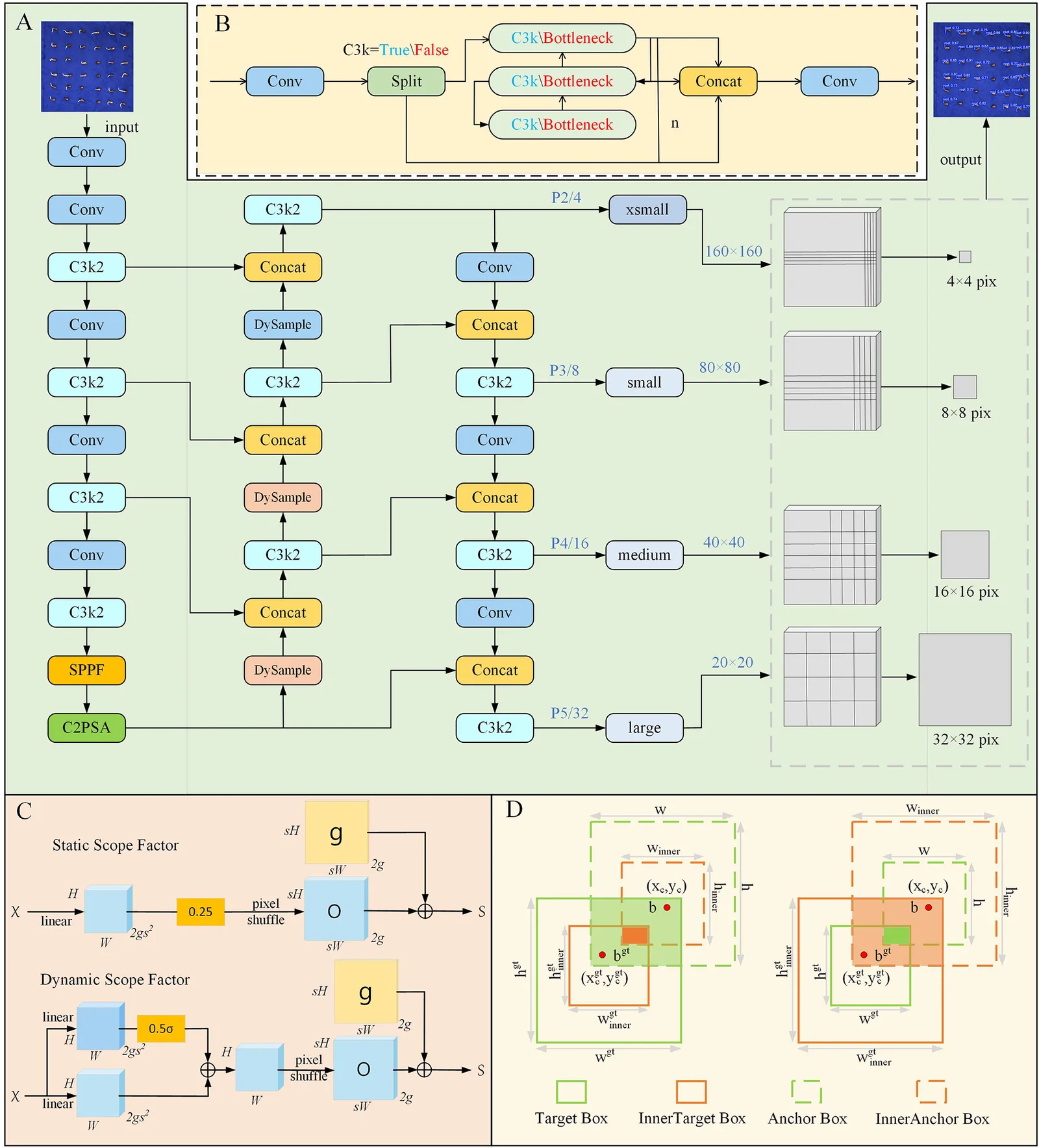
Network structure diagram of the improved model: **(A)** overall network structure; **(B)** principle of C3k2; **(C)** sampling point generator in Dysample; **(D)** overview of Inner-IoU.

The overall network architecture of the improved model is illustrated in **Figure 6**.

### 2.6 Ablation tests based on the improved modules

Ablation testing is a core method in scientific research used to evaluate the relationship between the role and performance of system components. It works by progressively removing, adding, or modifying system components (such as algorithmic modules, network layers, or data features) and observing the resulting changes in system performance, thereby determining the contribution of each component. To verify the effectiveness of each aforementioned improvement, we trained models obtained at each step using the same dataset and identical hyperparameters. Specifically, we conducted an ablation study on the improved experiments by sequentially adding a tiny object detection layer (S-YOLO11-seg) to the Neck part of the initial model (YOLO11-seg), replacing the upsampler nn.Upsample in the Neck layer with DySample (SD-YOLO11-seg), and introducing Inner-IoU for IoU optimization (SDI-YOLO11-seg). The parameters of each model recorded from this process are detailed in **Table 4** below.

**Table 4:** Performance metrics comparison of the improved models.

Model	mAPbox 50	mAPmask 50	Params (M)	FLOPs (G)	FPS (f/s)
YOLO11-seg	0.952	0.877	2.83	5.18	151.79
S-YOLO11-seg	0.958	0.931	3.17	13.30	134.04
SD-YOLO11-seg	0.961	0.935	3.19	13.38	114.34
SDI-YOLO11-seg	0.961	0.937	3.19	13.38	121.49

After adding the tiny object detection layer to the initial model, the mAPbox50 and mAPmask50 of the model showed significant improvement, increasing by 0.6% and 5.4% respectively. This indicates that the tiny object detection layer greatly contributes to enhancing the detection accuracy of the model. The Params and FLOPs increased by 0.34M and 8.12G respectively, likely because the model now predicts more anchor boxes per grid cell for high-resolution feature map processing, leading to a substantial increase in regression and classification computations within the detection head. On this basis, to further enhance the model’s capability for detecting small targets and its instance segmentation performance, we replaced the original nn.Upsample with the dynamic upsampler DySample. This resulted in additional increases of 0.3% and 0.4% in mAPbox50 and mAPmask50, respectively, with only minor increases in Params and FLOPs. Finally, to improve the model’s generalization capability and reduce the missed detection rate for densely arranged radicles in the late germination stage, we introduced Inner-IoU to optimize the original IoU, which further raised mAPmask50 by 0.02% while Params and FLOPs remained virtually unchanged. As shown in **Figure 7**, after implementing the above three improvements, the final model’s detection accuracy shows a clear enhancement compared to the initial model, with mAPbox50 and mAPmask50 increasing by 0.9% and 6.0% respectively, while maintaining relatively low model complexity.

**Figure 7:**
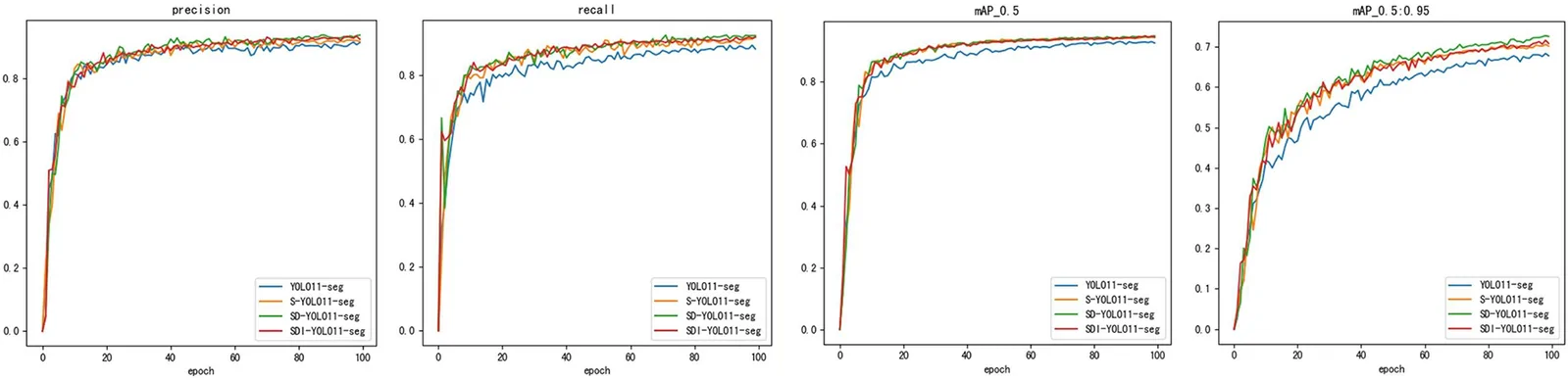
Performance metrics comparison of the improved models.

In order to more intuitively compare the detection and segmentation performance among the various models and validate the improvement of the final model compared to the initial model, inference predictions were conducted on images selected at specific intervals: one seed germination image every 8 hours during the 16–64 hour period of the germination stage, and one seedling growth image every 12 hours during the 48–192 hour period of the seedling stage. The comparative results are shown in **Figures 8**, **9**. As can be observed from the figures, when using YOLO11-seg for detection, a significant number of missed detections occurred during the early germination and emergence stages. After adding the tiny object detection layer, missed detections were substantially reduced, but false detections such as duplicate detections noticeably increased. After replacing the upsampler with the dynamic DySample, both missed and duplicate detections showed improvement. Finally, by introducing Inner-IoU for IoU optimization, the final SDI-YOLO11-seg model demonstrated a significant performance enhancement in detection and segmentation compared to YOLO11-seg. This model exhibited very few missed or duplicate detections for cotton radicles and cotyledons across all time periods, essentially meeting the accuracy requirements for cotton seed radicle detection and segmentation in this study.

**Figure 8:**
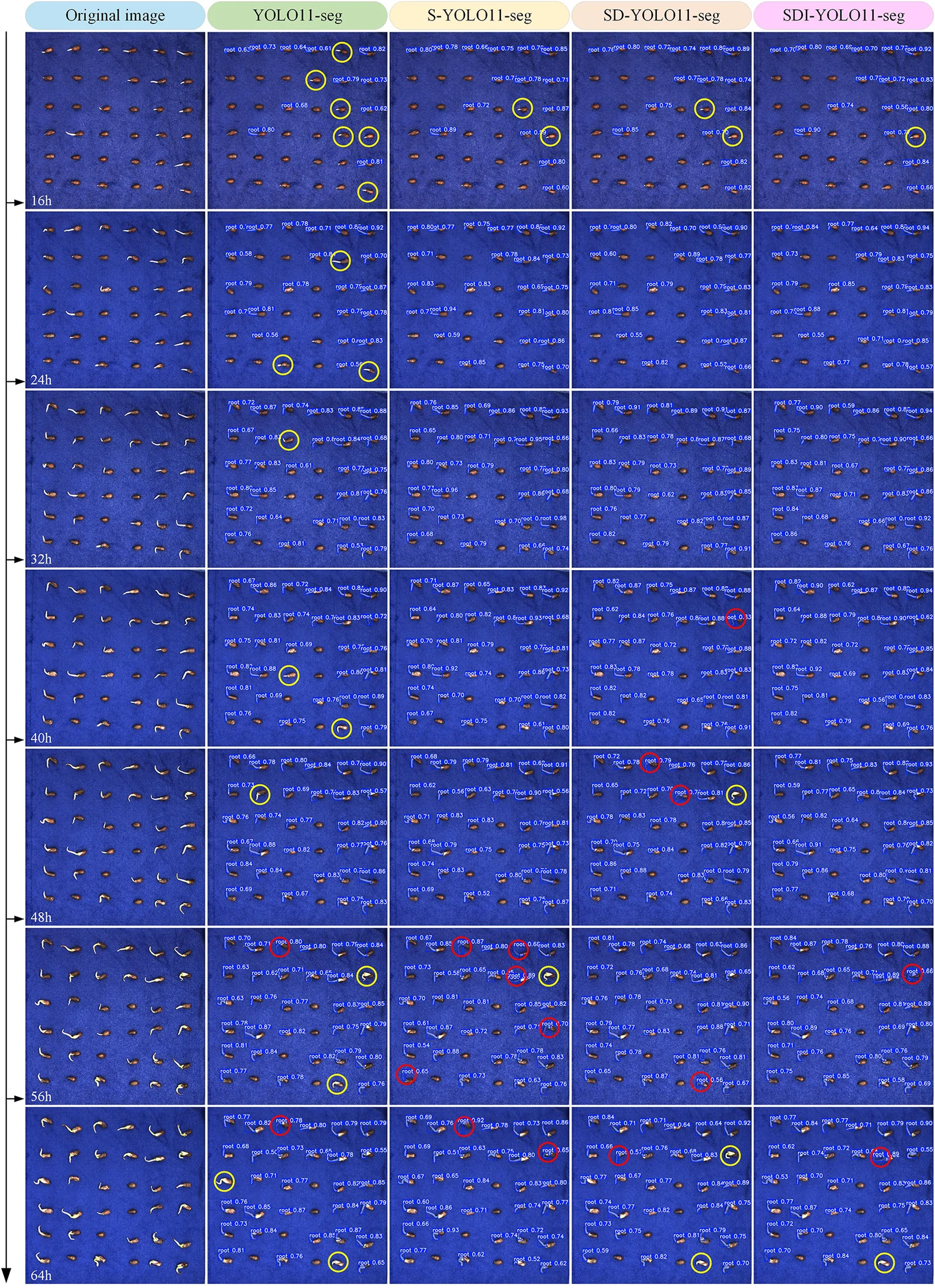
Improved model visualization of radicle detection results.

**Figure 9:**
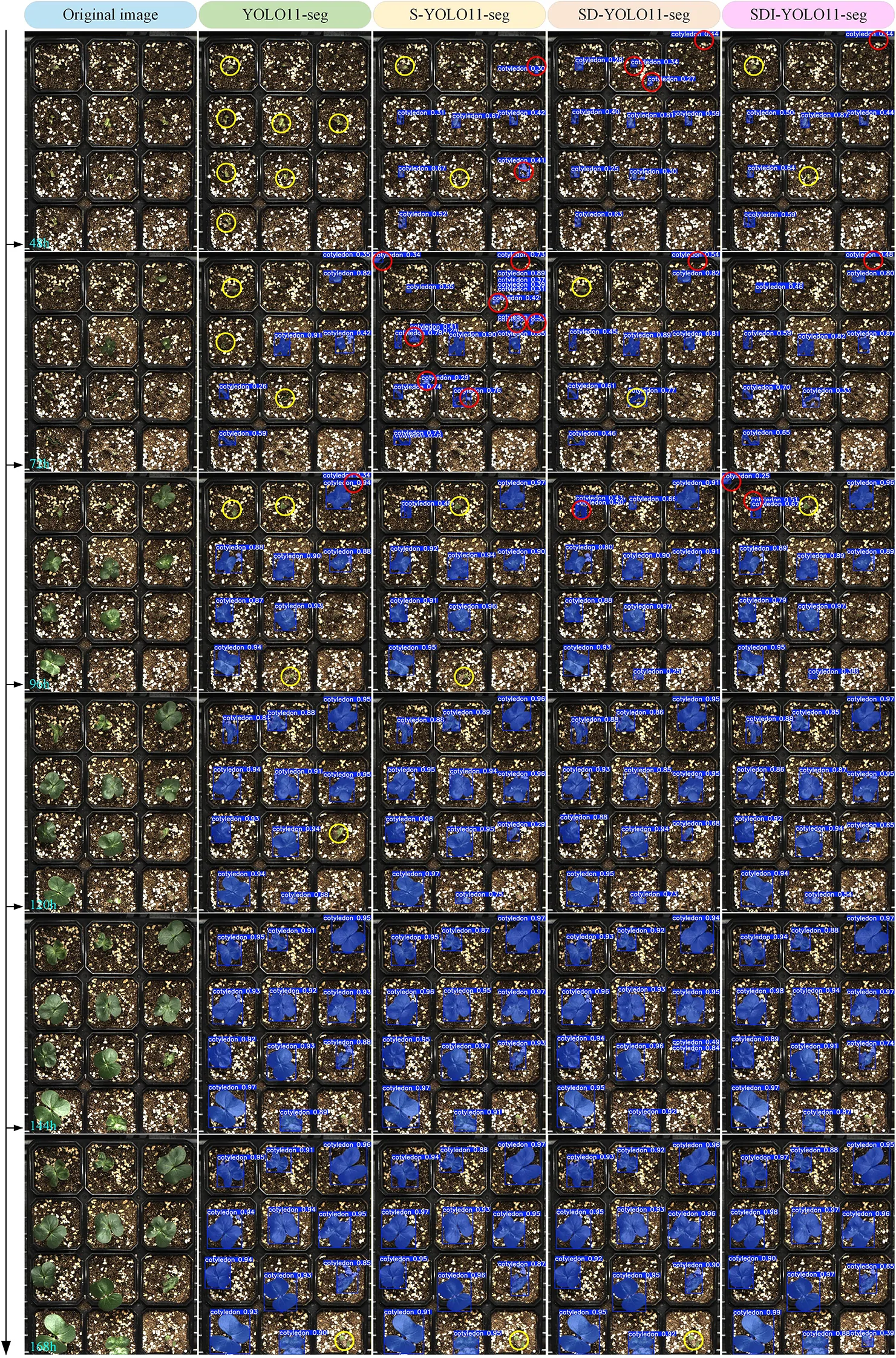
Improved model visualization of cotyledon detection results.

### 2.7 Accuracy analysis of the improved model

To validate the accuracy of the cotton radicle length and cotyledon area measurement method proposed in this study, the open-source image processing software ImageJ was used to manually annotate each radicle and cotyledon in the images. A pixel-to-physical unit conversion factor was established based on the scale bar to convert pixel distances and areas into actual physical units. A total of 16 randomly selected images, comprising 156 radicles and 119 cotyledons, were used as samples. The algorithm model was employed to measure each cotton radicle and cotyledon, and the measurement results were compared with those obtained from ImageJ through linear fitting analysis, as shown in **Figure 10A**. As seen in **Figure 10B**, the coefficients of determination (R²) for the two sets of data obtained by this method and ImageJ measurements are 0.97021 and 0.99344, respectively. This indicates a high degree of consistency between the two early-growth vigor indicators for cotton derived from this method and the ImageJ measurements, confirming that the method can provide reliable data support for subsequent salt tolerance analysis of different cotton varieties during the germination stage.

**Figure 10:**
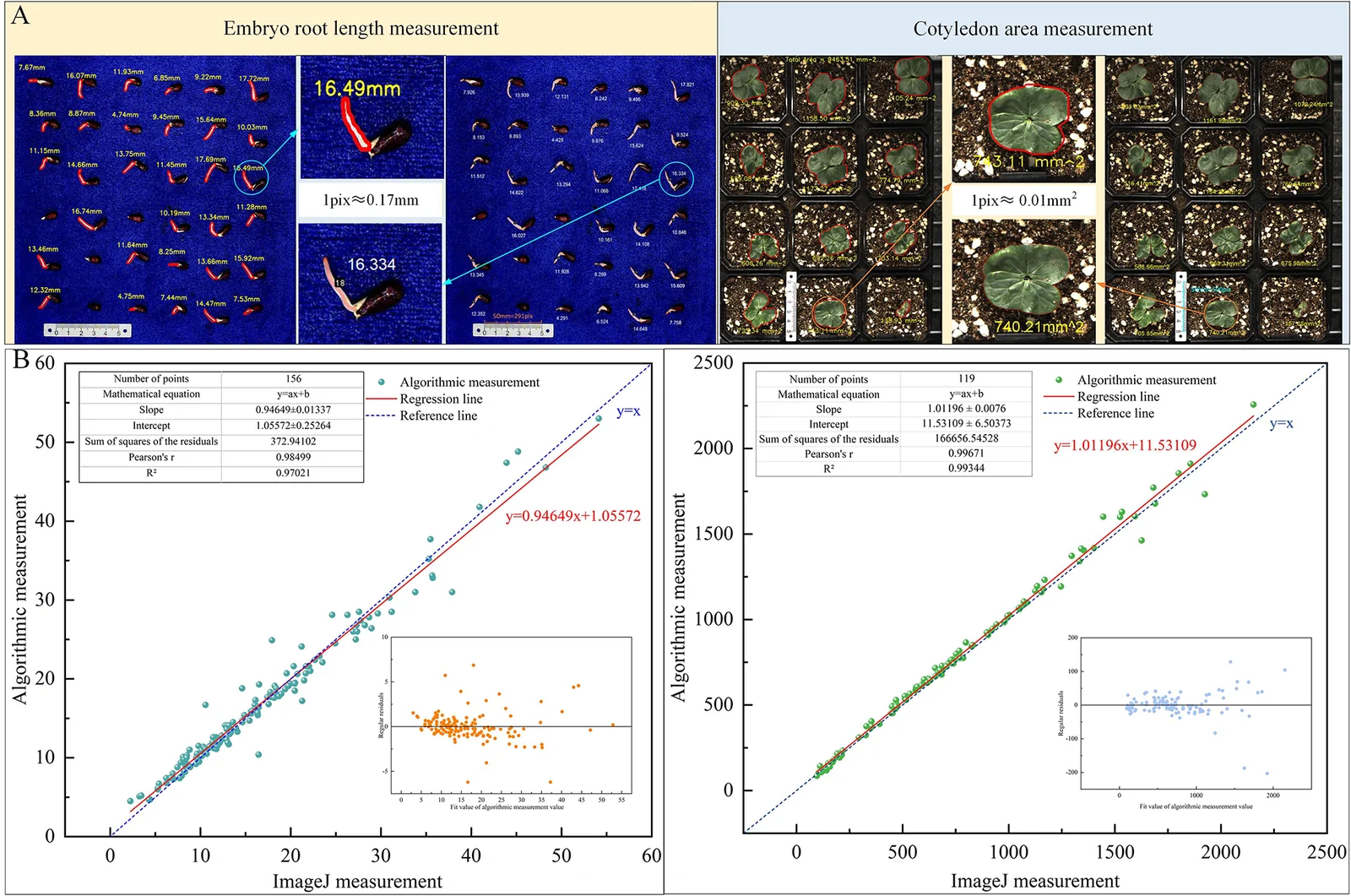
Accuracy analysis of the improved model: **(A)** measurement of radicle length and cotyledon area; **(B)** linear fitting analysis.

### 2.8 Experimental design

To evaluate the salt tolerance of different cotton varieties during the germination and seedling stages, we employed NaCl solutions with six concentration gradients (0, 50, 100, 150, 200, and 250 mmol/L) to simulate a saline stress environment. Growth vigor indicators during the germination and seedling stages for each variety under different salt stress levels were statistically analyzed to identify differences in salt tolerance. The NaCl solutions for the experiment were prepared using analytical grade sodium chloride from Sinopharm Chemical Reagent Co., Ltd. and deionized water. The cotton varieties used were Jinken 1565 (bred by the Cotton Research Institute of the Xinjiang Academy of Agricultural and Reclamation Sciences), Jinken 1775 (co-bred by the Xinjiang Academy of Agricultural and Reclamation Sciences and Huazhong Agricultural University), Tahe 2 (bred by Xinjiang Tarim River Seed Industry), and Xinluzao 64 (bred by Xinjiang Hexin Technology). These varieties are all major cultivars in southern and northern Xinjiang, making them representative. The entire experiment was conducted in two phases: a hydroponic germination stage trial and a soil-based seedling stage trial. Other experimental parameters and the specific workflow are detailed in **Figure 11**.

**Figure 11:**
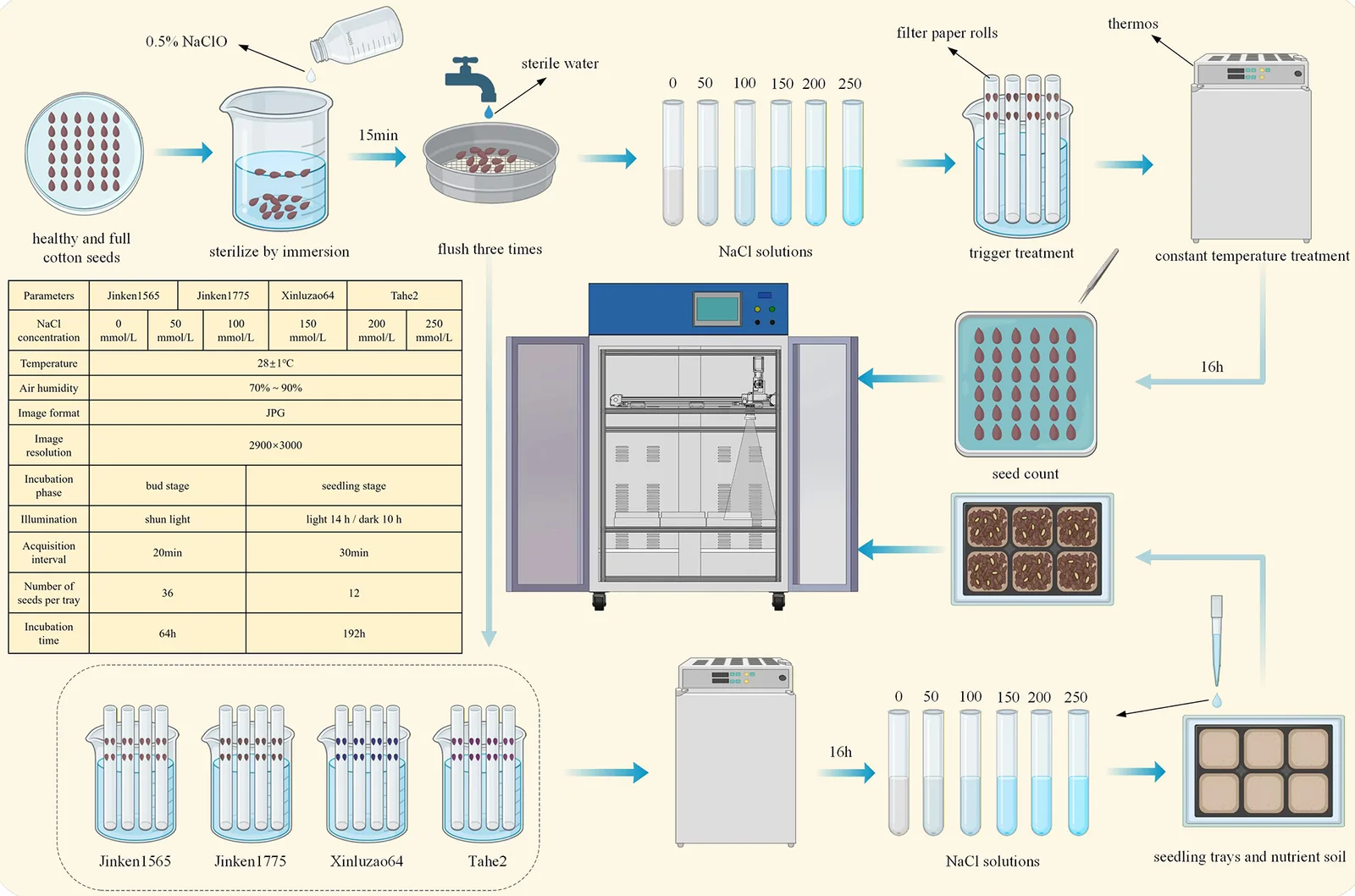
Experimental process of salt stress trials for four Xinjiang cotton varieties.

All data were analyzed statistically using SPSS 26.0 software. For indicators such as seedling emergence rate and cotyledon area across different crop varieties treated with varying NaCl concentrations, a normality test (Shapiro-Wilk test) and an equality of variance test (Levene’s test) were first conducted; when these assumptions were met, a one-way ANOVA was performed, followed by *post-hoc* multiple comparisons using the Tukey HSD method. When the data did not meet the assumptions of normality or equal variance, the Kruskal-Wallis non-parametric test and Dunn’s *post-hoc* test were employed. The significance level for differences was set at p < 0.05.

To evaluate the uniformity of growth vigor among individual seedlings within each treatment group, the coefficient of variation (CV) was calculated as the ratio of the standard deviation to the mean for each vigor indicator across three biological replicates. A lower CV value indicates more consistent growth among individuals, reflecting better population-level stability under the given stress condition. Under the control condition (0 mmol/L NaCl), where no salt stress is imposed, the coefficient of variation primarily reflects the measurement consistency of the monitoring system itself, including image acquisition stability, illumination uniformity across the culture area, and the precision of the automated positioning system. As such, the control-group CV serves as a baseline assessment of equipment performance. Under salt stress conditions, increases in CV relative to the control reflect the combined effects of biological variability in stress response among individual seedlings.

### 2.9 Selection of growth vigor indicators for the cotton germination and seedling stage

In seed germination experiments, the germination rate serves as an intuitive indicator for assessing seed quality and growth potential, while the germination potential and germination index are core metrics for evaluating seed germination vigor and stress resistance (Rajabi Dehnavi et al., 2020). To accurately assess the germination vigor of different cotton varieties, we used the detected number of radicles to represent the number of germinated seeds. The following formulas were applied to calculate the seed germination rate, germination potential, and germination index (Equations 2–4):

(2) $$Germination\;rate(\%)=(\frac{N_{t}}{N})\times 100\%$$

(3) $$Germination\;energy(\%)=(\frac{N_{48}}{N})\times 100\%$$

(4) $$Germination\;index=\sum (G_{t}/D_{t})$$

where Nt represents the number of seeds germinated at the t-th hour, N denotes the total number of seeds cultivated, N48 is the number of seeds germinated by the 48th hour, Gt is the number of seeds germinated within t hours, and Dt is the corresponding cultivation time. Additionally, the radicle length over continuous time periods was recorded. Its growth rate directly reflects seed vigor, which was used to assess the salt tolerance of each variety at the germination stage.

During the cotton cotyledon stage, the cotyledons serve as the primary nutritional organs before the formation of the first three true leaves. The size of the cotyledons is related to the photosynthetic efficiency of the plant. The cotyledon area is a key indicator for evaluating early growth vigor in cotton and an important component of the early Leaf Area Index (LAI) (Izzo et al., 2020). To maximize light capture efficiency and increase the light-receiving area, the two cotyledons of cotton seedlings during this stage exhibit a horizontally expanding growth morphology, driven by the combined effects of light-mediated cell expansion and hypocotyl mechanical thrust. This flat growth pattern makes them particularly suitable for area extraction using top-view images. Therefore, to accurately evaluate the growth vigor of different cotton varieties at the seedling stage, we quantified the cotyledon area of each seedling and the total leaf area for each experimental group, thereby assessing the seedling-stage growth vigor across different treatment groups.

## 3 Results and discussion

### 3.1 Comparison of germination stage growth vigor among different cotton varieties

To more intuitively compare the radicle growth vigor of the four cotton varieties under different levels of salt stress, this paper utilized the constructed SDI-YOLO11-seg model to perform inference detection on the captured germination process images of each cotton variety. The radicle length for each experimental group at 64 h was extracted, and the measurement results are shown in **Figure 12**.

**Figure 12:**
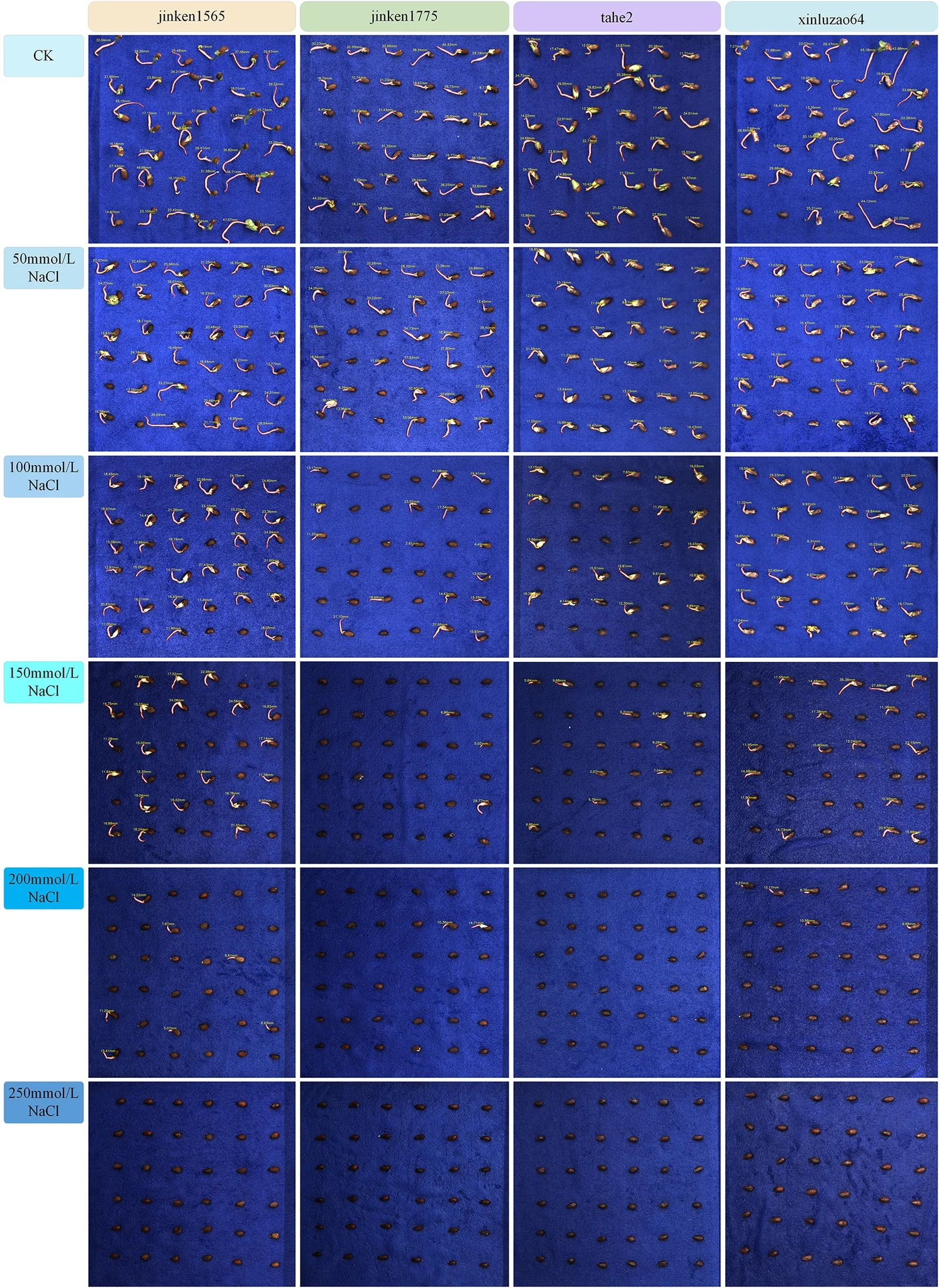
Visualization of cotton radicle length detection based on SDI-YOLO11-seg.

To compare the early growth vigor of the four cotton varieties and evaluate their differential salt tolerance under varying levels of salt stress, we recorded the number of germinated seeds and the germination rate at 4-hour intervals within the first 48 hours after initiating image acquisition. The results at the 48-hour mark were selected to represent the germination potential. The statistical outcomes are presented below.

**Supplementary Figure 1** illustrates the dynamic changes in germination rates of the four cotton varieties under treatments with different concentrations of NaCl solution. Experimental data show that in the control group without salt stress, all varieties exhibited excellent germination capacity: Jinken 1565 and Tahe 2 reached a 100% germination rate at 64 h, Jinken 1775 reached 97.72%, and Xinluzao 64 reached 88.89%. When the NaCl concentration increased to 100 mmol/L, significant differentiation in salt tolerance among the varieties emerged: the germination rates of Jinken 1775 and Tahe 2 plummeted to 52.78%, while Jinken 1565 and Xinluzao 64 maintained relatively high germination levels (88.89% and 86.11%, respectively), indicating stronger tolerance to salt stress. As the salt concentration further increased to 150 mmol/L, the germination rates of all varieties declined significantly, with Jinken 1775 and Tahe 2 dropping to 11.11% and 25%, respectively, demonstrating higher salt sensitivity. When the concentration reached 200 mmol/L, the germination rates of all varieties fell below the physiological threshold, with Tahe 2 even losing its germination ability completely. Under extreme salt stress at 250 mmol/L, no seed germination was observed for any variety, indicating that this salt concentration exceeded the tolerance threshold of all four varieties. **Supplementary Figures 2A**,**B** further quantify the differences in salt tolerance among the varieties through germination potential and germination index. Under low-salt conditions (≤50 mmol/L), there were no significant differences in the germination indicators among the varieties. However, when the salt stress intensity reached ≥100 mmol/L, the germination potential of Jinken 1565 and Xinluzao 64 was 68.2% and 61.4% higher than that of Jinken 1775, respectively, and their germination indices were 1.8 to 2.3 times higher. This difference was particularly pronounced in the 150 mmol/L treatment group. The experimental data indicate that Jinken 1565 and Xinluzao 64 possess more effective mechanisms for adapting to hyperosmotic environments during the germination stage.

To more intuitively compare the growth capacity of the four cotton varieties under different levels of salt stress, we utilized the constructed SDI-YOLO11-seg model to perform inference detection on the captured seed germination process images, extracting the radicle length for each experimental group at 64 hours. **Supplementary Figure 3** presents the distribution characteristics, central tendencies, and dispersion of radicle lengths for the four cotton varieties under varying salt stress concentrations using box plots. The results indicate that under the same salt stress conditions, significant differences exist in radicle length among the varieties, reflecting a clear genotype-specific nature of their salt tolerance. Based on the data measured by the model, a detailed analysis reveals the following: Under different salt concentration treatments, the radicle lengths of Jinken 1565 were 28.15 mm (0 mmol/L), 22.02 mm (50 mmol/L), 20.68 mm (100 mmol/L), 18.92 mm (150 mmol/L), and 13.27 mm (200 mmol/L). Its box plot distribution is concentrated with low dispersion, indicating that this variety can maintain relatively good growth stability under different salt stresses, demonstrating strong salt tolerance. The average radicle lengths of Xinluzao 64 under corresponding salt concentrations were 25.52 mm, 17.78 mm, 16.00 mm, 15.62 mm, and 9.23 mm. At low to medium concentrations (50–100 mmol/L), its average radicle length was close to that of Jinken 1565. Although lower than Jinken 1565 at high concentrations (150–200 mmol/L), it was still significantly higher than that of Jinken 1775 and Tahe 2, indicating a certain level of salt tolerance. In contrast, the average radicle lengths of Jinken 1775 under 0 mmol/L and 50 mmol/L NaCl treatments were 21.09 mm and 20.02 mm, respectively, suggesting some stability in low-salt environments. However, at concentrations of 100 mmol/L and above, the radicle length decreased significantly, indicating higher sensitivity to medium and high salt environments and a lower salt tolerance limit. Tahe 2 maintained some stability at 100 mmol/L, but when the salt concentration increased to 150 mmol/L and above, its average root length decreased by approximately 44.6%. Under 200 mmol/L conditions, it essentially reached zero, indicating weak tolerance to high salt levels, with severe inhibition of radicle growth at that point.

Based on the comprehensive analysis of the aforementioned germination-stage growth vigor indicators, the preliminary screening results for salt tolerance are as follows: the order of salt tolerance strength at the germination stage for the four varieties is Jinken 1565 > Xinluzao 64 > Jinken 1775 > Tahe 2. This ranking reflects the overall performance across all NaCl concentration gradients (0–200 mmol/L), integrating germination rate, germination potential, germination index, and radicle length. The differences between non-adjacent varieties in this ranking (e.g., Jinken 1565 vs. Jinken 1775) were statistically significant at concentrations ≥100 mmol/L (p < 0.05), while differences between adjacent varieties were significant at higher concentrations (150–200 mmol/L).

The coefficient of variation (CV) further revealed the uniformity of germination and radicle growth under salt stress (**Supplementary Table 1**). Under the control condition (0 mmol/L NaCl), the CV values of radicle length for all four varieties ranged from 5.2% to 6.0%, indicating satisfactory measurement consistency of the monitoring system. Under high NaCl concentration (200 mmol/L), Jinken 1565 exhibited the lowest radicle length CV (8.9%), indicating the most uniform radicle growth among individuals. In contrast, Tahe 2 displayed a substantially higher radicle length CV (22.3%) at 200 mmol/L, reflecting greater inter-individual variability. Xinluzao 64 showed moderate uniformity (radicle length CV: 12.1% at 200 mmol/L), while Jinken 1775 presented relatively high variability (18.5% at 200 mmol/L). Notably, no germination was observed at 250 mmol/L for these varieties, precluding CV calculation. These CV trends suggest that Jinken 1565 not only maintains greater average radicle growth but also exhibits superior population uniformity under salt stress, supporting its robust salt tolerance during the germination stage.

### 3.2 Comparison of seedling stage growth vigor among different cotton varieties

During the cotton germination stage, tolerance is primarily passive, relying on basic osmotic adjustment, resulting in generally weak salt tolerance. In the cotyledon stage, active adaptation mechanisms are enhanced, with synergistic improvements in salt tolerance through root barriers, ion transport, and antioxidant systems. Consequently, differences in salt tolerance exist between the germination and seedling stages of the same cotton variety. To compare the differences in salt tolerance among the four cotton varieties at the seedling stage, we recorded the emergence rate and cotyledon area at 12-hour intervals during the period from cotyledon emergence after transplantation until the control group developed its first true leaf (within 192 h). The results detected by the algorithm are shown in **Supplementary Figure 4** below.

**Supplementary Figure 5** shows the changes in the emergence rate of the four cotton varieties under different concentrations of NaCl solution. According to the experimental results, Jinken 1775 and Xinluzao 64 exhibited good salt tolerance during the cotyledon stage, as their emergence rates did not significantly decrease with increasing NaCl concentration. Statistical analysis results indicated that under treatment with 250 mmol/L NaCl, the emergence rate of Jinken 1775 and Xinluzao 64 (91.67%) was significantly higher than that of Jinken 1565 and Tahe 2 (50%) (p < 0.05, one-way ANOVA).

**Supplementary Figure 6** illustrates the changes in the total cotyledon area of the four varieties across the six experimental groups over time. As the concentration of the NaCl solution increased, the total cotyledon area for each variety gradually decreased within the same cultivation period, indicating that the effect of NaCl solution on cotyledon growth during the seedling stage of the four cotton varieties is consistent with its effect on the emergence rate: the higher the solution concentration, the stronger the inhibitory effect. As shown in **Supplementary Figure 6**, under low to medium salt stress (≤150 mmol/L), the total cotyledon area of Jinken 1775 and Xinluzao 64 remained at relatively high levels. Within the NaCl concentration range of 0–150 mmol/L, the total leaf area of Jinken 1775 at 192 h was significantly higher than that of Tahe 2 (p < 0.05), whereas no significant difference was observed between Jinken 1775 and Xinluzao 64 under most concentration treatments (p > 0.05). At 192 h, their total cotyledon areas were 18046.49, 15362.07, 14897.22, 10206.34, 6760.05, 6562.15 mm² and 16583.87, 14528.26, 12246.88, 10221.81, 8105.31, 5624.63 mm², respectively. Under these conditions, the cotyledon growth of Jinken 1775 slightly surpassed that of Xinluzao 64. However, under high salt stress, no significant difference in cotyledon area was observed between these two varieties. For Jinken 1565, the total cotyledon area at 192 h was 13356.93, 8782.28, 9095.35, 3564.03, 2316.35, 2193.57 mm², respectively. Its cotyledon area dropped sharply under medium to high salt stress, indicating relatively poor resistance to high-salinity environments during the seedling stage. The cotyledon growth of Tahe 2 remained at a consistently low level across all six experimental groups, demonstrating significantly weaker salt tolerance during the seedling stage compared to the other three varieties.

Based on a comprehensive comparison of the emergence rate and cotyledon area of the four cotton varieties, the order of salt tolerance strength at the seedling stage is Jinken 1775 > Xinluzao 64 > Jinken 1565 > Tahe 2. This ranking is primarily supported by statistical test results for the seedling emergence rate and cotyledon area at the 250 mmol/L NaCl treatment (p < 0.05, one-way ANOVA with Tukey HSD *post-hoc* test), where the differences between the tolerant varieties (Jinken 1775, Xinluzao 64) and the sensitive varieties (Jinken 1565, Tahe 2) were most pronounced. At lower concentrations, the differences between adjacent varieties in the ranking were not always statistically significant. Subsequent studies will further monitor the salt tolerance thresholds and stress-responsive enzyme activities across different crop varieties to elucidate the variations in salt tolerance among these varieties.

The CV analysis of seedling emergence rate and cotyledon area provided additional insights into varietal uniformity under severe salt stress (**Supplementary Table 2**). Under the control condition, the CV values of cotyledon area ranged from 4.2% to 5.5% across the four varieties, further confirming the reliability of the image-based measurement system. At 250 mmol/L NaCl, Jinken 1775 and Xinluzao 64 exhibited the lowest CV values for both emergence rate (5.2% and 5.8%, respectively) and cotyledon area (8.6% and 9.5%, respectively), indicating highly consistent seedling growth among individuals. Conversely, Tahe 2 showed the highest CVs (emergence rate: 18.5%; cotyledon area: 23.4% at 250 mmol/L), reflecting substantial heterogeneity. Jinken 1565 also displayed relatively high CVs (emergence rate: 12.8%; cotyledon area: 17.3% at 250 mmol/L), suggesting less uniform seedling vigor under extreme stress. These findings demonstrate that lower CV values in Jinken 1775 and Xinluzao 64 correspond to better uniformity of seedling vigor, complementing the mean-based assessments and providing a comprehensive evaluation of their salt tolerance.

### 3.3 Limitations and future perspectives

Due to limited experimental conditions and the absence of field validation, all experiments in this study were conducted within a controlled-environment incubator. While this setup allowed for precise control of temperature, humidity, light conditions, and salt stress concentration, it failed to simulate factors such as the heterogeneity of field soils in Xinjiang, microbial communities, and climatic variability. Therefore, the determined salt tolerance ranking requires validation through field trials in natural saline-alkali soils. Additionally, the agronomic traits analyzed in this study are not comprehensive. Currently, only early growth indicators such as germination rate, germination potential, germination index, emergence rate, and cotyledon area have been assessed, while later agronomic traits including plant height, number of fruit branches, boll weight, and fiber quality have not yet been included. Salt tolerance represents a comprehensive, multi-stage, and multi-trait phenotype. Hence, a holistic evaluation should be conducted in the future by integrating phenotypic data spanning the entire growth period. Furthermore, the range of cultivars selected in this study is limited, as only four major cultivars grown in Xinjiang were tested, resulting in a small sample size. Therefore, it is necessary to expand the cultivar range in future research to establish a more reliable salt tolerance evaluation system.

Additionally, the uniformity of the internal space within this system’s incubator has not yet been verified through systematic testing. Although the forced air circulation design helps reduce spatial gradients, systematic testing of the spatial distribution of temperature, humidity, light, and CO_2_ concentration should be conducted in the future to further confirm the equivalence of experimental conditions across different culture zones, thereby enhancing comparability between biological replicates and the reproducibility of the results.

This study primarily focuses on the quantification of morphological indicators such as radicle length and cotyledon area—parameters that exhibit low dependence on color information. However, for phenotypic analysis tasks involving color sensitivity or color intensity (e.g., chlorophyll content estimation, diagnosis of nutrient deficiency, disease lesion identification, or detection of stress-induced color changes), the consistency of the camera’s color response will be a critical factor influencing measurement accuracy. Therefore, in future system optimizations, a standard color chart will be integrated into the image acquisition module for periodic color calibration, and a color correction model will be developed to mitigate color biases arising from factors such as variations in illumination and camera aging, thereby ensuring the accuracy and cross-temporal comparability of color-related phenotypic data.

It should be noted that transformer-based real-time detectors such as RT-DETR (Zhao et al., 2024) were not included in the primary model comparison. This is because RT-DETR is designed exclusively for object detection and does not provide native instance segmentation capability, rendering metrics such as mAPmask50 inapplicable. Moreover, even from a detection-only perspective, RT-DETR’s parameter count (approximately 20–42 M) and computational complexity (60–136 G FLOPs) are substantially higher than those of the lightweight YOLO-series models evaluated herein (Ultralytics, 2025), making it unsuitable for the resource-constrained, real-time deployment scenario of our monitoring system. Should an official instance segmentation variant of RT-DETR become available, it would merit consideration in future comparative studies.

## 4 Conclusion

To rapidly and accurately assess the early growth vigor of Xinjiang cotton varieties and investigate the impact of varying salt stress levels on cotton seed vigor during the germination and seedling stages, this study developed a Full-Time Series Crop Growth Vigor Monitoring System. This system integrates seed germination cultivation, environmental control, automated irrigation, and image acquisition. Hydroponic germination-stage and soil-based seedling-stage experiments were conducted on four Xinjiang cotton varieties. Germination and growth images were captured over 64 hours for the germination stage and 192 hours for the seedling stage. Based on this, a labeled dataset documenting the cotton seed germination and seedling growth processes was constructed.

To meet the detection requirements for radicles and cotyledons, this study proposed an improved instance segmentation model named SDI-YOLO11-seg, based on the YOLO11-seg framework. This model achieved higher detection accuracy by incorporating a tiny object detection head, replacing the upsampler, and optimizing the IoU calculation. Compared to the original model, SDI-YOLO11-seg improved mAPbox50 and mAPmask50 by 0.9% and 6.0%, respectively. Utilizing this model, the study successfully measured cotton seed germination rate and seedling emergence rate. Through segmenting the contours of cotton radicles and cotyledons, the radicle length and cotyledon area were quantified based on the pixel count in the resulting binary images.

To validate the feasibility of the image processing and deep learning-based approach for screening salt-tolerant cotton varieties proposed in this study, hydroponic and soil cultivation experiments were performed on seeds of four Xinjiang cotton varieties using six different concentrations of NaCl solution. Vigor indicators, including germination rate, germination potential, germination index, emergence rate, and cotyledon area, were quantified using the SDI-YOLO11-seg model. By analyzing the germination and growth vigor of cotton seeds across 48 experimental groups, the following conclusions were drawn: Overall, NaCl solution exhibited a significant inhibitory effect on the growth vigor of all four cotton varieties during the germination and seedling stages, with the inhibitory effect strengthening as concentration increased. Furthermore, the experimental results revealed significant differences in salt tolerance among the same cultivars during the germination stage and the seedling stage: the order of salt tolerance strength during the germination stage was Jinken 1565 > Xinluzao 64 > Jinken 1775 > Tahe 2, based on significant differences in germination rate, germination potential, germination index, and radicle length at NaCl concentrations ≥100 mmol/L (p < 0.05). Whereas during the seedling stage, the order of salt tolerance strength was Jinken 1775 > Xinluzao 64 > Jinken 1565 > Tahe 2, based on significant differences in emergence rate and cotyledon area at 250 mmol/L NaCl (p < 0.05). Additionally, CV analysis revealed that Jinken 1565 exhibited superior growth uniformity during the germination stage, whereas Jinken 1775 and Xinluzao 64 demonstrated better uniformity during the seedling stage, providing additional dimensions for salt tolerance assessment beyond mean-based comparisons.

## Data Availability

The original contributions presented in the study are included in the article/**Supplementary Material**. Further inquiries can be directed to the corresponding author.
